# Transforming mental health services: a participatory mixed methods study to promote and evaluate the implementation of recovery-oriented services

**DOI:** 10.1186/s13012-014-0119-7

**Published:** 2014-09-10

**Authors:** Melissa M Park, Hiba Zafran, Janet Stewart, Jon Salsberg, Carolyn Ells, Suzanne Rouleau, Orly Estein, Thomas W Valente

**Affiliations:** School of Physical and Occupational Therapy, Faculty of Medicine, McGill University, Montréal, QC H3G 1Y5 Canada; Participatory Research at McGill, McGill University, Montréal, QC H3S 1Z1 Canada; Lady Davis Institute for Medical Research, Jewish General Hospital, Montreal, QC H3T 1E2 Canada; Centre de santé et services sociaux de la Montagne, Montreal, QC H2V 1 K5 Canada; Department of Family Medicine, Participatory Research at McGill, McGill University, Montreal, QC H3S 1Z1 Canada; Department of Medicine and Biomedical Ethics Unit, Faculty of Medicine, McGill University, Montreal, QC H3A 1X1 Canada; Institute of Community and Family Psychiatry, Montreal, QC H3T 1E4 Canada; Ordre des Ergotherapeutes du Quebec, Montreal, QC H3A 2A6 Canada; Centre de santé et services sociaux Cavendish, Montreal, QC H4W 2 T5 Canada; Department of Preventive Medicine, Keck School of Medicine, University of Southern California, Los Angeles, CA 90089-9175 USA

**Keywords:** Recovery, Applied policy, Mixed methods, Social network analysis, Ethnography, Narrative-phenomenology, Participatory research, Integrated knowledge translation

## Abstract

**Background:**

Since 2007, the Mental Health Commission of Canada has worked collaboratively across all provinces to publish a framework and strategy for recovery and well-being. This federal document is now mandated as policy for implementation between 2012 and 2017. The proposed strategies have been written into provincial health plans, hospital accreditation standards, and annual objectives of psychiatric departments and community organizations. The core premise is: to empower persons with mental illness and their families to become participants in designing their own care, while meeting the needs of a diverse Canadian population. However, recovery principles do not come with an implementation guide to fit the variability of different local contexts. How can policy recommendations and accreditation standards be effectively tailored to support a diversity of stakeholder values? To our knowledge, there is little evidence indicating the most effective manner to accelerate the uptake of recovery-oriented services among providers in a given/particular mental health treatment setting.

**Methods/Design:**

This three-year Canadian Institute of Health Research Partnership in Health System Improvement and The Rx&D Health Research Foundation (HRF) Fostering Canadian Innovation in Research study (2013 to 2017) proposed participatory approaches to implementing recovery principles in a Department of Psychiatry serving a highly diverse Canadian and immigrant population. This project will be conducted in overlapping and recursive phases: I) Conduct formative research to (a) measure the current knowledge and attitudes toward recovery and recovery-oriented practices among service providers, while concurrently (b) exploring the experiential knowledge of recovery service-users and family members; II) Collaborate with service-users and the network-identified opinion leaders among providers to tailor Recovery-in-Action Initiatives to fit the needs and resources of a Department of Psychiatry; and III) Conduct a systematic theory-based evaluation of changes in attitudes and practices within the service-user/service-provider partnership group relative to the overall provider network of the department and identify the barriers and supports within the local context.

**Discussion:**

Our anticipated outcome is a participatory toolkit to tailor recovery-oriented services, which will be disseminated to the Mental Health Commission of Canada and Accreditation Canada at the federal level, agencies at the provincial levels, and local knowledge end-users.

## Background

Severe mental illness (SMI) constitutes five out of ten leading causes of disability, remaining one of the most debilitating conditions facing global mental health, with high economic and ethical costs [1]. Over 1 million Canadians (3%) live with SMI with high direct ($4.7 billion) and indirect ($3.2 billion) economic costs [2]. This is exacerbated by the global phenomena of displacement of persons for economic and political reasons [3]. The dire situation and the fragmentation of mental health services in Canada led to the establishment of the Mental Health Commission of Canada (MHCC) in 2007, whose objective was to evaluate and develop a national mental health policy for Canada. Subsequently, the MHCC generated policy recommendations for recovery in a report titled ‘Toward Recovery and Well-Being: A Framework for a Mental Health Strategy of Canada’ [4], which culminated in the launch of Canada’s first national mental health strategy, ‘Changing Directions, Changing Lives: The Mental Health Strategy for Canada’ [5]. The national policy, while being rooted in the principles of recovery and well-being, intentionally lacks precise directives for implementation. Thus, the MHCC ultimately recognizes that the creation of a mental health system aligned with recovery-oriented services from a ‘fragmented patchwork of programs and services’ [4:13] requires policy that remains open for a range of regional governments and local institutions to adapt national policy to a diversity of needs as well as contextual supports and barriers.

National regulatory bodies, such as Accreditation Canada, provide incentives for integrating principles of recovery-oriented service into practice standards, which must be met by 2017. However, implementing recovery-oriented services will require more than acquiring a new skill set or behaviors. The recovery model creates a radical shift in the conceptualization of practice and ethics that conflict with traditional biomedical practice concepts and goals [6]. For example, the focus of recovery goals on self-management, choice, hope and transformation, contrast starkly with the traditional biomedical approach to mental illness, which stresses eradication and/or control of the disease. This recovery orientation also shifts responsibility and control of treatment from provider to patient [7], which can increase role ambiguity and threaten traditional status hierarchies. This emphasis on patient autonomy, and empowering service-users to make decisions based on their personal values, complicates attempts to create normative standards that fit the diversity of values, experiences and contexts of a range of service-users, and variability within one patient’s recovery trajectory. Thus, recovery-oriented services will require not only a transformation of practice standards, but also a transformation of biomedical practice, itself. As the MHCC [5:36] specifically underlines, ‘Experience in other countries and here at home tells us that it will take a sustained action on many fronts to truly shift culture and practice in the mental health system toward recovery and well being.’ Consequently, the recovery model of mental health is a radical or discontinuous innovation [8] that is unlikely to diffuse rapidly or easily within mental health treatment organizations. This raises a fundamental question: Can we transform the values and practices of a service organization by taking a participatory and relational approach to the design and implementation of recovery-oriented services?

To our knowledge, there is no implementation research to date on how to tailor broad policy objectives and new practice guidelines to support recovery-oriented services. In fact, intervention studies in public health comprise less than 10% of published studies. In health promotion studies that use outcome measures, pre-post measures are rarely used [9]. Even fewer studies examine the implementation process itself [10], although recent emphases on implementation underscore the importance of this topic (*e.g*., see: http://www.cepim.med.miami.edu, *Implementation Science*). Further, specific to developing recovery-oriented services, we do know that ‘mental health research would benefit from implementation studies that investigate how to involve stakeholders in changing practices, as well as how to create safe conditions for dialogue and collaborative processes’ [11]. In sum, there is little evidence indicating the most effective manner to accelerate the uptake of recovery-oriented services among providers in a mental health treatment setting. To address this gap, we are conducting a process evaluation to understand the creation, dissemination, and effects of our Recovery Promotion Program (RPP) toolkit, a package of theoretical and methodological strategies for promoting and evaluating recovery-oriented services.

### Research question and partners

In order to close the gap between national policy recommendations and accreditation standards and the self-identified needs in a local context, this three-stage interdisciplinary, mixed methodology research project will embed a participatory research project within an ethnography of everyday mental health practices at a Department of Psychiatry that serves a highly diverse population. Participatory research is an approach defined as ‘systematic inquiry with the collaboration of those affected by the issue under study for the purposes of action or change’ [12,13]. Our two-pronged approach consists of: (a) participatory implementation of emerging practice guidelines for recovery-oriented services; and (b) integrated knowledge translation (IKT) or the translation of the experiential knowledge of providers and service-users, as informed by family member perspectives.

**Figure 1:**
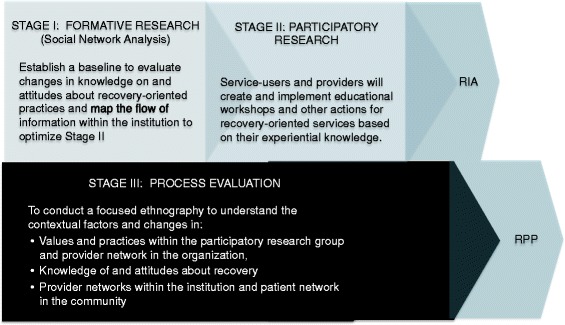
**Anticipated timeline of the research design: Methodology and sub-questions for each stage of the project.**

Our primary research question is: How can policy recommendations for, and standards of, recovery-oriented mental health services be effectively tailored to support a diversity of stakeholder values? The overall design of our project consists of three stages, which will unfold in an iterative and overlapping process across three years. Each stage is defined by a methodology and related sub-questions (see Figure 1).

To ensure that new knowledge is relevant to stakeholder groups, we set our primary research question with the support of a partnership formally established for this project with:

1. The Program Development Team of Accreditation Canada, a not-for-profit organization accredited by the International Society for Quality in Health Care, which has a mission to create standards that assure quality of patient-centered care, while remaining flexible enough to reflect the diversities of resources and priorities across organizations, regions, and provincial and territorial jurisdictions [14];
2. The providers and staff of a Department of Psychiatry located in a culturally diverse (over 100 different national origins, many of whom are first-generation immigrants to Canada) urban area, which is at an institution with a ‘care for all’ philosophy that underlines the diversity of interests, ethnocultural and religious values to which recovery must be customized;
3. AMI-Québec, a grassroots non-profit organization that provides support, education and advocacy services to family members of persons with mental illness; and
4. Formations Porte-Voix, the first and only national organization of advocacy run by and for persons living with a mental disorder, an organization that has key objectives to promote recovery and to translate knowledge to decision-makers.

Our intended outcome is a Recovery Promotion Program (RPP) toolkit, a package of theoretical and methodological strategies for promoting, tailoring and evaluating the implementation of recovery-oriented services.

### Mixed methods to innovate an RPP toolkit

In health promotion research, there are two common measures: dose and fidelity. Dose is the degree of program exposure or intensity of its delivery, and is measured during program implementation by documenting the amount of program material created and disseminated. Fidelity refers to the degree to which a program is implemented as planned. Documenting how a particular program was produced, and the decisions that influenced message production, is critical to implementing successful programs across a diversity of settings [10]. Unlike many evaluation studies, which treat the intervention as a ‘black box,’ we aim to develop a comprehensive understanding of how this treatment approach gets woven into the everyday fabric of systems processes. The RPP toolkit for user-designed implementation of innovations and its evaluation consists of: social network analysis, participatory approaches, and ethnographic methods.

### Theoretical framework: narrative & phenomenological

A theoretical framework is essential to the development and evaluation of health promotion programs, in specifying the mechanisms of change, the goals to be set, what behaviors will be changed, and how outcomes will be measured [10]. The methods involved in this study have been specifically chosen for their theoretical underpinnings as well as their integrated approach to evaluating particular relational processes within a specific context. A transformative mixed methods research and evaluation paradigm incorporates stakeholders in the design and implementation of research whose aim is to foster and evaluate change [15]. Thus, we have embedded the design and implementation of Recovery-in-Action Initiatives (RIA) within an overarching evaluation of the process and effects of a participatory approach to tailoring recovery-oriented care with stakeholders.

Based on our research question and the complexity of understanding the diversity of experiences, values and concerns of multiple stakeholders in a changing context, we chose a narrative-phenomenological (NP) analytic framework [16] for the following three reasons: First, it focuses attention on what matters to or is at stake for particular individuals around, often shared, events. Second, this framework is well suited to examine change over time in the experiences of vulnerable individuals living with chronic illness and clinical practice, as it was developed from a longitudinal (over a decade), person-centered, and multiple perspective ethnography of healthcare encounters in a culturally diverse and urban context [16–18]. Third, it has a philosophical grounding in phenomenological concepts and narrative theory while also providing specific analytic units that capture the larger structural constraints and resources that guide everyday action. These three analytic units are:

1. Discursive practices. Discursive practices are the master narratives, or genres, that enter into everyday language, and which guide actions. This is of interest as ‘Recovery,’ much like the narratives of Alcoholics Anonymous [19], has a particular structure that is learned through practice. In addition, there are genres in biomedicine [18] that guide clinical reasoning, often implicitly. We will document how recovery narratives enter into biomedical discursive practices and relate to actions.
2. Person-centered. Attention is paid to ‘particularities’ – of actions, persons, places, including the aesthetic qualities of experience [20–23], which are heightened during transformative moments.
3. Event-focused. Events are situations that are experienced as significant and which, through analysis, can make visible what is at stake to those persons involved [24]. Events, particularly those that deviate from the expected, often signal transformative moments.

Our narrative-phenomenological framework (NP) will inform data collection and analysis around both verbal and enacted narratives. Stories are particularly potent vehicles for understanding the experiences of others, supporting mutual understanding, and even, acting as change agents: stories create shifts in attitudes and values as listeners come to care about the experiences of others [25]. Thus, a narrative-phenomenological framework is consistent and supportive of the transformative agenda of our participatory approaches to implementation and research, where creating positive change or action occurs through the collaborative and equal involvement of stakeholders [26].

### Social network analysis

Social network analysis (SNA) is a set of theories and techniques used to understand how social relationships (*e.g*., friendship, advice seeking, reputation) influence behaviors [27–29]. Most relevant to the current study is the discovery of the importance of peer network influences on behavior change. This research has led to the development of peer-based interventions, which is the foundation of the implementation strategy we propose [30]. It is widely acknowledged that behavior change programs are more effective when designed and implemented by the intended audience [31]. For example, opinion leaders (OLs) identified using network data are highly engaged in promoting behavioral changes in many settings [32–34]. OLs are present in all organizations and are usually defined as informal leaders, not formal administrative leaders. OLs influence the knowledge, attitudes and behaviors of others in the community or organization, and diffusion occurs much more rapidly when initiated by OLs. In clinical settings, Lomas *et al*. [32] found significant effects using OLs to change practice. Given that recovery orientation is a radical innovation, using OLs to promote change is an essential ingredient to effective change management. In sum, the network data provide an aid to program implementation, and understanding how targeted behaviors spread in an organization.

### Identifying OLs

Network data can be used to identify these individuals who can be engaged as conduits or champions for successful behavior change efforts across the network [35]. Many prior studies have demonstrated that social network data may be collected quickly, reliably and cheaply in organizational settings. OLs are identified by selecting those in the network who receive the most nominations in response to a questions such as, ‘Who do you go to for advice about (topic of interest)?’ Individuals with the most nominations are identified as OLs, who are then engaged as champions or change agents in the new initiative or diffusion of information. To date, most SNA studies have identified experts in the system through peer nomination e.g., see [36,37]). It is clear from recent research, however, that the self-reported opinion leadership is weakly correlated to social network-identified OLs [38]. Curran *et al*. [37] recommend that investigators consider focusing on nominations based on communication, reflecting trust, as opposed to expertise, because trust may be an important element in effective use of opinion leaders. Trustworthiness is a distinct domain that taps communication flow; it is an assessment of symmetrical rather than hierarchical relationships [28]. This approach is particularly relevant to our study because we are interested in identifying individuals who are respected for their clinical acumen (expertise) and who are in personal communication with their peers (trusted).

### Intraorganizational communication (IC)

Provider network data will indicate who goes to whom for advice and discussion. Combined with data on providers’ recovery knowledge, attitudes and practices (RKAP), we can accurately describe how this new recovery approach spread through the provider networks, if it did. We can also assess the extent to which certain leaders spread these attitudes and behaviors in their personal networks. We are conscious that the network data may indicate that strategies and/or tactics other than working exclusively with opinion leaders may be more appropriate in this setting at this time [30].

### Participatory research

Participatory research (PR) is an approach in which researchers work in equitable partnerships with those affected by the research and/or those who must ultimately act on its results [12,13,39,40]. Its goals are to foster self-determination and social justice among affected populations while co-creating and translating new knowledge into action for change [12,41]. PR is a preferred approach to implementation research as it integrates end-users – those who must ultimately act on or benefit from a new policy or practice, such as service providers and users – throughout the implementation process [13,42]. In mental health, patient inclusion in this process is aligned with the principles of empowerment and management of recovery.

The PR approach will be used to tailor policy recommendations to a Department of Psychiatry by highlighting the salient concerns and integrating the practical knowledge of the contextual barriers and/or supports to recovery-oriented services. By definition, practical or experiential knowledge consists of what we have learned through accumulated experience. Experiential knowledge is often overlooked and underutilized in traditional health promotion programs. In contrast, user-design empowers and thus effectively engages users in the decision-making process [43]; therefore enhancing the relevance and applicability of the research to its end-users and increasing the likelihood of knowledge uptake and sustainability [39,41]. We will focus specifically on working with providers and service-users to draw from their experiential knowledge of recovery trajectories (service-users) and practice supports and constraints to implementing recovery services (providers) as they design Recovery-in-Action Initiatives. We anticipate the RIA to consist of a training workshop for the providers and staff at the Department of Psychiatry, and other emergent actions. For example, in a previous PR project, patient and provider members partnered outside of the group to present an anti-stigma campaign at AMI-Québec, entered other recovery-related research teams, and requested patient involvement in advisory committees [11].

### Ethnography

We will use ethnographic methods to monitor participatory processes, which are emergent and cannot be predetermined (*i.e*., no fidelity measures), in order to inform how future tailored programs to implement recovery can be accelerated and successfully replicated across a diversity of contexts. Ethnographic methods provide details or thick descriptions on the interaction between particular persons within particular contexts [44]. Our intent, however, is not to describe the ‘culture’ of biomedicine or of the institution as a set of values, attitudes, beliefs and patterns of behaviors. Rather, we define culture as what we do every day or ‘cultural practices’ (e.g. see [45,46]). Implementing recovery-oriented services will require a fundamental change in what providers do every day. For example, the principles of self-management and outcomes oriented to the patient’s hopes in recovery frameworks [47] will shift providers’ roles, responsibilities, and the construct of the therapeutic relationship. Thus, we will use ethnographic strategies to describe the transformation of clinical practice as a cultural system, itself.

Practices and transformations of practices are not easily accessed through collection of texts alone. Practices are embodied, patterns of habitual actions or habitus [48,49] that can also be evidenced as ‘common sense’ [50]. In contrast to psychometric observational scales, which calculate outcomes based on discrete behaviors, ethnographic participant observations allow us to enter into the habitual everyday routines of providers and service-users. Thus, over time, we are better situated to experience and understand when values, attitudes, actions of particular person change. In particular, reflexive attention to unexpected turns in events often point to transformational moments [51] and can be traced back to particular triggers or active ingredients of change [52]. Guided by the narrative-phenomenological framework [18], we examine how the actions and interactions of stakeholders change over time by systematically documenting changes in (a) discursive practices, (b) knowledge of and attitudes on recovery, and (c) the interactions during events.

### Project design

#### Stage-I: Formative evaluation to determine baseline on recovery-oriented services

In Stage-I, we will conduct a formative mixed methods research study to answer the following key sub-questions: (a) What is the social network and flow of information in the Department of Psychiatry?; and (b) What are the current knowledge, attitudes, and practices related to recovery-oriented services?

### Recruitment

The research team members presented the project, handed out consents and answered questions at provider and administration team meetings and a research forum at a Department of Psychiatry. Service-users were referred by providers and contacted by team members to go over consent process. All individuals consented individually and in private. Research ethics approval was received from the site of implementation prior to participant recruitment.

### Data collection

1. Recovery Knowledge Inventory (RKI) [53] for providers. The RKI was developed with and from stakeholder experiences (narratives) and assesses four domains: (a) roles and responsibilities in recovery, (b) non-linearity of the recovery process, (c) the roles of self-definition and peers in recovery, and (d) expectations regarding recovery;
2. Social Network Questionnaires to identify opinion leaders, based on trust as well as expertise in the provider network, gaps and bridges in IC, and support in patient networks; and
3. Focus groups with service-users (Department of Psychiatry) and family members (local community) to elicit collective narratives or shared experiences of what matters most to service-users and family members on recovery [16], which will be de-identified and fed-forward to the participatory group in Stage-II in order to maximize the integration of patient and family stakeholder values in the design of the RIA initiatives.

#### Stage-II: Participatory approaches to create Recovery-In-Action Initiatives (RIA)

In participatory research, stakeholders identify gaps, and we generated the following questions in collaboration with the Department of Psychiatry: (a) What are the gaps between the national standards on recovery-oriented services and local values, knowledge about, and attitudes on recovery?; (b) What are the barriers to and supports for recovery-oriented services?; and (c) What actions will we take to tailor national policy and accreditation standards to fit the local values, needs, and contextual factors in order to evolve our clinical practice for highest quality outcomes?

### Recruitment

We will recruit four network-identified provider opinion leaders and four service-users who participated in Stage-I focus groups.

### PR process

Consented PR group members will meet for 20 sessions, over 2.5 years. The transcripts of each audiotaped session will be de-identified and available to all PR members. The following proposed sequence and objectives, informed by research team experience [11,54,55], is:

1. Establish group safety and collaborative processes. Identify objectives for participating, describe overarching purpose, collaboratively establish expectations of confidentiality, and discuss differences in the research versus patient-provider contract (two sessions).
2. Explore and examine experiences. Provide password-protected access to a shared drive, in which de-identified transcripts from Stage-I (service-users, family member focus groups) will be stored. These transcripts will be read and discussed during the first session in order to generate key topics of concern related to recovery. Over the next sessions, participants will take turns telling and discussing personal stories related to the chosen recovery topics. The purpose is to build mutual understanding of each other’s experiences and to generate material on key topics of concern. All sessions will be audiotaped, transcribed, and participants identified only as provider, patient, or paire aidante certifiée (*i.e*., certified peer support worker). The transcriptions will be made available for analysis prior to, and key stakes identified and discussed in, subsequent sessions (six sessions).
3. Design of Recovery-in-Action Initiatives (RIA). Identified areas of concern related to recovery-oriented services and constraints and supports in the system will be ranked according to relationship to local values and needs, and will rank areas of concern, identify contextual factors that constrain and support recovery-oriented care, and create RIA initiatives, such as a training workshop in addition to other strategies or actions (six sessions).
4. Implementation. The RIA will be presented to our partners, including providers and staff of the Department of Psychiatry during Grand Rounds, family and caregivers of persons with SMI of AMI-Québec, persons with lived experience of mental illness with vested interest in research at Formations Porte-Voix, and the project development team of Accreditation Canada (four sessions).
5. Debrief and Closure. Lessons learned about the PR process will be agreed upon and consolidated for the final report (two sessions).

#### Stage-III: A systematic, theory-based ethnographic process evaluation

Effective process evaluation requires (a) multiple measures, (b) across multiple points in time, (c) from multiple perspectives. This assures our ability to analyze the success or failure of different media (or methods) to reach individual stakeholders. Our key sub-questions for Stage-III are: (a) What are the process and effects of a participatory action approach to design and implement programs tailored to local needs and values on recovery?; and (b) What are the contextual factors of the local setting that may be barriers or supports to knowledge translation?

### Data collection

It is essential that we monitor the participatory processes, which are emergent and cannot be predetermined (*i.e*., no fidelity measures) in order to inform how future programs can tailor and accelerate the uptake of recovery-oriented services across a diversity of settings. In order to understand the contextual factors of the local setting that may be barriers or supports to knowledge translation (e.g., see [56]) and to examine how the participatory approaches operated, we will use ethnographic methods across all stages, including:

1. Trained ethnographic participant observation. This is a central and often missing method to capture how initiatives enter into the everyday actions of organizational members. Informed by narrative-phenomenology, we will examine how recovery discourses, knowledge and attitudes enter into clinical practice and interact with experiences of stakeholders (service-users, families, providers, floor staff).
2. Focus groups and individual semi-structured interviews with providers and/or administrators. Stories are a vehicle for making sense of change and disruption and will be collected in group and individual format.
3. Scheduled reviews of the media and literature will be conducted by the research assistant for research, public campaigns, media, and/or events related to recovery and/or recovery-oriented services. This data will be logged and will be used to monitor for other influences within the local culture that could impact on changes in RKAP.
4. Attendance sheets will be logged to correspond with the following quasi-control groups determined by dose or amount of exposure to RIA initiatives: (a) Least exposure: no attendance at RIA workshop or other emergent initiatives; (b) Some exposure: attendance at either RIA or involvement in other emergent initiatives; (c) Moderate exposure: interview participation; (d) Increased exposure: focus group participation; and (e) Highest exposure: PR group participation.

Additionally, during Stage-II and Stage-III, we will collect data on the effect of the RPP toolkit:

1. RIA production (Stage-II): Attendance at PR sessions, transcripts of sessions, and intragroup communication (*e.g*., memos, email, PR member field notes and/or reflective diaries).
2. Re-administration of the Recovery Knowledge Inventory (RKI) (Stage-II and Stage-III) for providers.
3. Re-administration of the Social Network Questionnaires for providers (Stage-II and Stage-III) to monitor IC and for service-users and family members (Stage-III) for changes in support systems.

### Data analyses: mixed methods

Collaborative mixed methods research is dependent ‘as much on the researchers’ capacities to learn though joint effort and to construct joint meaning as on their expertise in conventional data collection and analysis techniques’ [57]. Thus, our analysis requires on-going reflexive dialogue [58] in order to utilize and develop ‘different specialties in reflective terms… It is a question of acquiring a suitable combination of similarities and differences in the collaborative undertaking’ [59]. We will create an audit trail of how our analytic process occurs since the process of collaborative knowledge generation rather than ‘obfuscation’ [57] will be central in the provision of study results. Our strategies consist of:

1. Reflection team meetings. We will meet systematically during the analytic process and invite stakeholders from the Department of Psychiatry who are experts in the organizational system, in mixed methods and in narrative-phenomenological ethnography ‘to consume the data and offer their feedback’ [60]. In these team meetings, explicitly stating the research paradigm, including methodology, epistemology and researchers’ moral-ethical stance [60,61], will be critical to rigour and to maximizing the strengths and collaborative process of our interdisciplinary team [62].
2. Ongoing iterative analysis. The narrative-phenomenological framework will focus analysis on the structural or discursive practices (recovery policy, cultural genres) and events that emerge in social interaction from the perspective of particular persons. Given the overarching mixed methods research design, and multiple positionalities of several of the researchers, we will reflexively make explicit and draw on our multiple theoretical perspectives and epistemologies during analysis, as well as on published research, to make supportive links to existing and emerging evidence and develop working hypotheses. Iterative analysis includes strategies such as maintaining detailed descriptions and field notes, collaboratively creating an audit trail for both the qualitative and quantitative analyses, having members of the research team with pertinent expertise in the different methods, and the use of triangulation of the different forms of data collected, including details of discrepant situations or stories. As part of this iterative analysis, and in line with overall transformative and embedded nature of this study, emergent analyses and results will be shared with stakeholders at the Department of Psychiatry for their feedback and input.
3. We will use validity criteria specific to mixed methods, including summarizing and presenting the results and inferences for each of the research objectives and stages separately, with attendant critiques, prior to combining, comparing and/or contrasting the meaning of the overall results. The combination of all the results (including negative, contradictory or resistant situations or stories) into a consistent, theoretically coherent meta-inference that is supported by expert consensus and the literature to date is called ‘integrative efficacy’ [63]. Expert review of the final RPP by the study partners, *i.e*., the whole research team, hospital administrators, department chiefs, community organization, and Accreditation Canada could give feedback on the integrative efficacy and utility of the study analyses.
4. In our reflective team meetings, we will also use emotions as validity checkpoints to monitor our ‘internal dialogue’ [60]. Thus, the reflections recorded in ethnographer’s (participant-observer’s) field notes and researcher’s diaries will be used to monitor and render explicit the trail of how emotions, experience and intellectual leanings may have affected the interpretation and analysis process e.g. see also [61]). During collaborative meetings, these diaries and notes will be shared with invited stakeholders to further the cycles of reflection, and to promote the inter-subjective analysis dimensions of a participatory project, as well as promote a critical reflexive stance towards the research study.
5. Finally, integrative correspondence relates the meta-inferences to the original research purpose [63], or: what are the results good for? Were the RIAs useful and transformative? Do the proposed RPP and measured outcomes align with the MHCC’s framework and mental health strategy? Is it cost-effective and feasible? The transferability anticipated and deliberately aimed for in this transformative research includes ecological transferability [15,63] and the provision of evidence and a toolkit for other psychiatry organizations who intend to transform services.

### Anticipated outcomes

There are several significant outcomes anticipated from this study. First, we will provide a deep understanding, both narrative and quantitative, of how a complex new treatment regime is understood and potentially adopted within a healthcare organization. Second, we will test methods for accelerating clinical practice change to treat a significant medical condition. Third, we will provide a toolkit to disseminate findings and potentially impact clinical treatment on a wider scale throughout Canada. Fourth, we anticipate being able to document the utility of this mixed methods approach to achieve better research and clinical outcomes, since the approach is inclusive and transformative.

### Trial status

We received ethics approval from the Research Ethics Committee where the research is being conducted, since it is the main site and key investigators are affiliated there. At the time of writing, the research is at the first stage.

## Discussion

### Study limitations

Resistance to change is a well-documented organizational reality, which we have aimed to address through the participatory and integrated nature of the study design. The foremost limitation to this study is that it only includes one practice site, so is perhaps a case study rather than a clinical trial. This limitation to breadth, however, is offset by the ability to have participant observers make deep and lasting recordings of how an organization can transform itself. Given the local, emergent and participatory nature of this study, we anticipate the following potential limitations: (a) the participatory research process is dependent on the amount of time the identified OLs and self-selected research participants will need to establish trust and learn to work together; and (b) the RPP toolkit will need to be implemented and evaluated in different contexts to re-verify its utility and validate its generalizability.

### Ethical considerations

Combining diverse stakeholder perspectives requires additional ethical considerations in PR processes. Perceived and experienced differences in traditional patient-provider roles can lead to increased vulnerability. Service-users and providers may feel restrained to speak openly in front of each other, particularly about challenging or negative experiences. To address these additional vulnerabilities, we have put the following precautionary measures in place: First, service-users will not be currently receiving services from the providers participating in PR. Second, certified peer support workers will facilitate sessions (JSt, OE). Certified peer support workers are persons with lived experience of mental illness who are trained to provide support in line with the recovery model. We have found that their experiences as service-users and training as support workers position them as mediators and translators of both patient and/or provider perspectives during divergent moments. Third, research team investigators who are dually positioned clinician-researchers with expertise in leading and teaching group dynamics (SR, HZ) and/or experienced with PR processes (MP, JSa) will co-moderate the PR sessions. Fourth, we will hold the PR groups in a context different from where participants provide or receive services. Although group processes are emergent and highly variable, we have sequenced the sessions in a step-wise process to strengthen collaboration and action outcomes. The study was approved by the Bureau d’Éthique de la Recerche, Hôpital general juif/Research Ethics Office, Jewish General Hospital.

### Feasibility

Feasibility can be a central concern of mixed methods grants: different epistemologies can endanger team processes [64]. PR processes also involve complex and sensitive collaboration, which can be complicated by real and perceived hierarchical differences when academics, clinicians, service-users, partner up. Throughout the development of this proposal, we have taken a ‘distributed leadership’ stance that reflects the strengths that each team member brings with his or her methodological expertise and experience with PR, SNA, Ethnography, and Narrative-Phenomenology. Combined, we have a focus on ‘connectedness’ from both structural (SNA) and relational, first-person perspectives (Participatory, Ethnographic), including clinical expertise teaching and leading group processes. In addition to bringing complementary methodological perspectives, we have the added benefit of team members’ dual positioning as provider-researchers, patient-researchers, and/or patient-provider-researchers.

## Abbreviations

(HRF): Rx&D health research foundation
(SMI): Severe mental illness
(MHCC): Mental health commission of Canada
(RPP): Recovery promotion program
(IKT): Integrated knowledge translation
(RIA): Recovery-in-action initiatives
(NP): Narrative-phenomenological conceptual framework
(SNA): Social network analysis
(OLs): Opinion leaders
(IC): Intraorganizational communication
(RKAP): Recovery knowledge, attitudes, and practices of recovery
(PR): Participatory research
(RKI): Recovery knowledge inventory

## References

[1] Knapp M, Mangalore R, Simon J (2004). The global costs of schizophrenia. Schizophr Bull.

[2] Citizens for Mental Health: **Backgrounder: Mental Illness in Canada.** In Canadian Mental Health Association; 2003:2.

[3] Bhugra D, Minas IH (2007). Mental health and global movement of people. Lancet.

[4] Mental Health Commission of Canada: **Toward recovery and well-being. A framework for a Mental Health Strategy for Canada.** In Calgary, AB: Author; 2009:127.

[5] Mental Health Commission of Canada: **Changing directions, changing lives: The mental health strategy for Canada.** In Calgary, AB: Author; 2012:152.

[6] Slade M (2009). 100 Ways to Support Recovery: A guide for Mental Health Professionals. Rethink.

[7] Marshall SL, Crowe TP, Oades LG, Deane FF, Kavanagh DJ (2007). A review of consumer involvement in evaluations of case management: consistency with a recovery paradigm. Psychiatr Serv.

[8] Rogers EM (1995). Diffusion of Innovations.

[9] Israel BA, Schulz AJ, Parker EA, Becker AB (1998). Review of community-based research: assessing partnership approaches to improve public health. Annu Rev Public Health.

[10] Valente TW (2002). Evaluating Health Promotion Programs.

[11] Schwartz R, Estein O, Komaroff J, Lamb J, Myers M, Stewart J, Vacaflor L, Park M (2013). Mental health consumers and providers dialogue in an institutional setting: A participatory approach to promoting recovery-oriented care. Psychiatr Rehabil J.

[12] Green LW, George MA, Daniel M, Frankish CJ, Herbert CP, Bowie WR, O'Neill M (1995). Study of Participatory Research in Health Promotion. Book Study of Participatory Research in Health Promotion.

[13] Parry D (2009). A Guide to Researcher and Knowledge-User Collaboration in Health Research. Canadian Institutes of Health Research (CIHR).

[14] Accreditation Canada~Agrément Canada (2010). Accreditation Canada Strategic Plan 2010–2013. Book Accreditation Canada Strategic Plan 2010–2013.

[15] Mertens DM (2009). Transformative Research and Evaluation.

[16] Mattingly C (2010). The Paradox of Hope: Journeys through a Clinical Borderland.

[17] Lawlor MC, Mattingly C (2006). Resituating Cultural Competence: An ethnographic and longitudinal study. Book Resituating Cultural Competence: An ethnographic and Longitudinal Study.

[18] Lawlor MC, Mattingly C (2000). Boundary crossing: an ethnographic and longitudinal study. University of Southern California.

[19] Peacock JL, Holland DC (1996). The narrated self: life stories in process. Ethos.

[20] Mattingly C, Lawlor MC (2001). The fragility of healing. Ethos.

[21] Stoller P (1989). Fusion of the Worlds: an Ethnography of Possession among the Songhay of Niger.

[22] Csordas TJ, Hefner R (2002). The rhetoric of transformation in ritual healing. Body/Meaning/Healing.

[23] Csordas TJ, Weiss G, Haber HF (1999). Embodiment and cultural phenomenology. Perspectives on Embodiment: The Intersections of Nature and Culture.

[24] Jackson M (2005). Existential Anthropology: Events, Exigencies and Effects.

[25] Garro LC, Mattingly C, Mattingly C, Garro LC (2000). Narrative as construct and construction. Narrative and the cultural construction of illness and healing.

[26] Walter M, Walter M (2009). Participatory Action Research. Social Research Methods.

[27] Moore KA, Peters RH, Hills HA, LeVasseur JB, Rich AR, Hunt WM, Young MS, Valente TW (2004). Characteristics of opinion leaders in substance abuse treatment agencies. Am J Drug Alcohol Abuse.

[28] Valente TW (2010). Social Networks and Health: Models, Methods, and Applications.

[29] Wasserman SFK (1994). Social Network Analysis: Methods and Applications.

[30] Valente TW (2012). Network interventions. Science.

[31] Slater MD, Kelly K, Edwards R, Plested B, Thurman PJ, Keefe T, Lawrence F, Henry K (2006). Combining in-school and participatory, community-based media efforts: Reducing marijuana and alcohol uptake among younger adolescents. Health Educ Res.

[32] Lomas J (1998). Social capital and health: Implications for public health and epidemiology. Soc Sci Med.

[33] Flodgren G, Parmelli E, Doumit G, Gattellari M, O’Brien MA, Grimshaw J, Eccles MP (2011). Local opinion leaders: effects on professional practice and health care outcomes (Review). Cochrane Lib.

[34] Valente TW, Pumpuang P (2007). Identifying opinion leaders to promote behavior change. Health Educ Behav.

[35] Valente TW, Davis RL (1999). Accelerating the diffusion of innovations using opinion leaders. Ann Am Acad Polit Soc Sci.

[36] Peters RH, Moore KA, Hills HA, Young MS, LeVasseur JB, Rich AR, Hunt WM, Valente TW (2005). Use of opinion leaders and intensive training to implement evidence-based co-occurring disorders treatment in the community. J Addict Dis.

[37] Curran GM, Thrush CR, Smith JL, Owen RR, Ritchie M, Chadwick D (2005). Implementing research findings into practice using clinical opinion leaders: Barriers and lessons learned. Jt Comm J Qual Patient Saf.

[38] Iyengar R, Van den Bulte C, Valente TW (2010). Opinion leadership and contagion in new product diffusion. Market Sci.

[39] Jagosh J, Macaulay AC, Pluye P, Salsberg J, Bush PL, Henderson J, Sirett E, Wong G, Cargo M, Herbert CP, Seifer SD, Green LW, Greenhalgh T (2012). Uncovering the benefits of participatory research: implications of a realist review for health research and practice. Milbank Quart.

[40] Macaulay AC, Commanda LE, Freeman WL, Gibson N, McCabe ML, Robbins CM, Twohig PL (1999). Participatory research maximises community and lay involvement. Br Med J.

[41] Cargo M, Mercer S (2008). The value and challenges of participatory research: strengthening its practice. Annu Rev Public Health.

[42] Graham ID, Tetroe J (2007). How to translate health research into effective healthcare action. Healthc Q.

[43] Carr-Chellman A, Savoy M, Jonassen DH (2004). User-design research. Handbook of Research on Educational Communications and Technology.

[44] Geertz C (1973). The Interpretation of Cultures.

[45] Holland D, Lachicotte W, Skinner D, Cain C (1998). Identity and Agency in Cultural Worlds.

[46] Ortner SB (1999). The Fate of 'Culture': Geertz and Beyond.

[47] Canada MHCo (2009). Toward Recovery and Well-Being. A Framework for a Mental Health Strategy for Canada.

[48] Bourdieu P (1977). Outline of a Theory of Practice.

[49] Bourdieu P, Wacquant LJD (1992). An Invitation to Reflexive Sociology.

[50] Geertz C (1983). Common Sense as a Cultural System. Local Knowledge: Further Essays in Interpretive Anthropology.

[51] Park M: **Beyond calculus: Apple-apple-apple-ike and other embodied pleasures for a child diagnosed with autism in a sensory integration based clinic.** *Disabil Stud Quart Special Topic Autism Neurodivers* 2010, **30**.

[52] Park M (2012). Pleasure, throwing breaches, and embodied metaphors: tracing transformations-in-participation for a child with autism to a sensory integration-based therapy session. OTJR Occup Participat Health.

[53] Bedregal L (2006). The recovery knowledge inventory: assessment of mental health staff knowledge and attitudes about recovery. Psychiatr Rehabil J.

[54] Salsberg J, Macaulay A, Straus S, Tetroe J, Graham ID (2013). Linkage and Exchange Interventions. Knowledge Translation in Health Care: Moving from Evidence to Practice.

[55] Pelletier J-F, Lesage A, Delorme A, Macaulay AC, Salsberg J, Vallée C, Davidson L (2011). Feature ~ user-led research: a global and person-centered initiative. Int J Mental Health Promot.

[56] Glasgow RE, Emmons KM (2007). How can we increase translation of research into practice? Types of evidence needed. Annu Rev Public Health.

[57] Shulha LM, Wilson RJ, Tashakkori A, Teddlie C (2003). Collaborative Mixed Methods Research. Handbook of Mixed Methods in Social & Behavioral Research.

[58] Morgan DL (2007). Paradigms lost and pragmatism regained. Methodological implications of combining qualitative and quantitative methods. J Mix Methods Res.

[59] Alvesson M, Sköldberg K (2009). Reflexive Methodology.

[60] Leavy P (2009). Method Meets Art: Art-Based Research Practice.

[61] Davies J, Spencer D (2010). Emotions in the Field.

[62] Denzin N, Lincoln Y (2000). Handbook of Qualitative Research.

[63] Teddlie C, Tashakkori A (2009). Foundations of Mixed Methods Research: Integrating Quantitative and Qualitative Approaches in the Social and Behavioral Sciences.

[64] Curry LA, O’Cathain A, Clark VLP, Aroni R, Fetters M, Berg D (2012). The role of group dynamics in mixed methods health sciences research teams. J Mix Methods Res.

